# The burden of X-linked retinitis pigmentosa (XLRP) on patient experience and patient-reported outcomes (PROs): findings from the EXPLORE XLRP-2 study

**DOI:** 10.1038/s41433-024-03546-8

**Published:** 2025-01-07

**Authors:** Francesco Parmeggiani, Michel Weber, Dominique Bremond-Gignac, Avril Daly, Tom Denee, Marjolein Lahaye, Andrew Lotery, Nabin Paudel, Markus Ritter, Enrique Rodríguez de la Rúa, Ygal Rotenstreich, Eeva-Marja Sankila, Katarina Stingl, Jacqueline Van Denderen, Katalin Pungor

**Affiliations:** 1 Department of Translational Medicine, University of Ferrara, Ferrara, Italy; 2 Center for Retinitis Pigmentosa of Veneto Region - ERN-EYE Network, Camposampiero Hospital, Azienda ULSS 6 Euganea, Padua, Italy; 3 Ophthalmology Department, University Hospital Centre (CHU) de Nantes, Nantes, France; INSERM UMR 1089, University of Nantes, CHU de Nantes, Nantes, France; 4 Ophthalmology Department, Necker Enfants Malades University Hospital, AP-HP, Paris Cité University, Paris, France; 5 INSERM, UMRS1138, T17, Sorbonne Paris Cité University, Centre de Recherche des Cordeliers, Paris, France; 6 Retina International, Dublin, Ireland; 7 Janssen-Cilag B.V., Breda, The Netherlands; 8 Faculty of Medicine, University of Southampton, Southampton, UK; 9 Department of Ophthalmology, Medical University of Vienna, Vienna, Austria; 10 Department of Ophthalmology, University Hospital Virgen Macarena, Seville, Spain; 11 Department of Surgery, Ophthalmology Area, University of Seville, Seville, Spain; 12 RiCORS-REI, Instituto de Salud Carlos III (RD21/0002/0011), Seville, Spain; 13 The Goldschleger Eye Institute, Sheba Medical Center, Tel Hashomer, Israel; 14 Ophthalmology Department, School of Medicine, Faculty of Medical and Health Sciences, Tel Aviv University, Tel Aviv, Israel; 15 Sagol School of Neuroscience, Tel Aviv University, Tel Aviv, Israel; 16 Department of Ophthalmology, University of Helsinki and Helsinki University Hospital, Helsinki, Finland; 17 University Eye Hospital, Center for Ophthalmology, University of Tübingen, Tübingen, Germany; 18 Center for Rare Eye Diseases, University of Tübingen, Tübingen, Germany; 19 Janssen-Cilag GmBH, Neuss, Germany

**Keywords:** Quality of life, Retinal diseases, Hereditary eye disease

## Abstract

**Background/aims:**

X-linked retinitis pigmentosa (XLRP) is considered one of the most severe forms of retinitis pigmentosa (RP), accounting for 5–15% of all RP cases and primarily affecting males. However, the real-world humanistic impacts of this disease on patients are poorly investigated, especially with respect to burdens faced by patients with varying disease severities.

**Methods:**

EXPLORE XLRP-2 was an exploratory, multicentre, non-interventional study. A retrospective chart review was conducted to collect clinical/demographic data, including XLRP clinical stage (mild, moderate or severe). Cross-sectional surveys were used to gather experiences directly from patients by validated and modified patient-reported outcomes.

**Results:**

176 patients with XLRP caused by retinitis pigmentosa GTPase regulator (*RPGR*) gene mutation were enrolled, of whom 169 were included in analyses. 81% of patients were male, mean (SD) age was 39.3 (17.61) years, and 20 adolescents were included. Mean age (SD) at genetic confirmation was 33.4 years (17.98), and the mean duration (SD) from initial symptoms to genetic diagnosis was 16.4 (15.66) years. Compared with patients with mild disease, patients with severe XLRP are more likely to experience difficulties with functioning in low luminance, depression, unemployment, productivity issues, mobility and daily activities.

**Conclusion:**

This is the first real-world study to collect data directly from patients on the burden of XLRP and to correlate that burden with disease stage. As a result, several areas of significant burden, especially for patients with severe disease, have been identified that should provide focus for future public policies and therapeutic prospects.

## Introduction

Retinitis pigmentosa (RP), the most frequently occurring form of inherited retinal diseases (IRD), causes progressive vision loss, generally leading to legal or total blindness by the fourth or fifth decade of life. X-linked retinitis pigmentosa (XLRP) is considered one of the most severe forms of RP, accounting for 5–15% of all cases [1–6]. As an X-linked disorder, XLRP primarily affects males, but female carriers can also experience a range of mild-to-severe progressive visual impairments [7–9].

More than 70% of XLRP cases are caused by mutations in the retinitis pigmentosa GTPase regulator (*RPGR*) gene, resulting in irreversible degeneration of photoreceptors [3, 5, 10]. The earliest symptom is usually nyctalopia (night blindness), which may start in childhood. As the disease progresses, patients experience tunnel vision as their visual field deteriorates, while acuity of central vision also declines. Continued deterioration of visual function results in legal blindness at a median age of 45 years [1].

To date, no studies have specifically explored social, economic, psychological and emotional burdens in people with XLRP, leaving a significant gap in our understanding of how patients are affected by this disease, particularly during its different stages [6, 11, 12]. As new treatments are emerging, it is becoming more important to understand the impact of XLRP on patients’ lives.

To increase our understanding of burdens faced by people with XLRP, the EXPLORE XLRP-2 study was undertaken to collect real-world data directly from patients and from their medical records across multiple countries. Medical charts were reviewed, and patients were surveyed with the primary objective of exploring the relationship between the clinical stages of XLRP and the associated clinical, individual, and societal levels of burden. This manuscript summarises the primary outcomes of the study.

## Methods

EXPLORE XLRP-2 was a non-interventional study conducted in 23 centres across 10 countries (Austria, Belgium, England, Finland, France, Germany, Israel, Italy, the Netherlands and Spain). Each participating site identified male and female patients who: (1) were aged ≥12 years at screening; (2) had XLRP confirmed by a retina specialist and had a predicted disease-causing sequence variant in *RPGR* confirmed by genetic testing; (3) were able and willing to give informed consent or assent (with the guidance of a legally acceptable representative, as applicable); and (4) had no participation (currently or previously) in a gene therapy trial. No formal sample size calculation was performed. The sample size of approximately 150 to 200 patients was primarily based on pragmatic considerations, such as disease rarity and ability to enrol enough eligible patients per disease stage for this descriptive and exploratory study.

Insights from clinical experts and patient experts were collected via advisory board meetings to guide development of the study design.

The study received local ethical committee approval at each study site (Austria: 1088/2022; Belgium: B3222022000832; England: 307690; Finland: 48/2022; France: 22.02349.000114; Germany: 839/2021BO1, 2022-200343-BO-bet; Israel: 0391-21-SOR, 9186-22-SMC; Italy: 1026/2021/Oss/AOUFe; the Netherlands: 21.177/VS; Spain: PI2022051) and adhered to the tenets of the Declaration of Helsinki. All participants gave informed consent.

Staging of vision was performed at sites based on information from the last visit (vision status of the better-performing eye). Patients were rated ‘mild’, ‘moderate’ or ‘severe’ based on visual acuity and visual field diameter measurements (Supplementary Table 1) in accordance with the ‘Visual Standards – Aspects and Ranges of Vision Loss’ report from the International Council of Ophthalmology [13].

Data were collected from two sources. One source was cross-sectional self-administered surveys. Feedback on survey questions was collected via interviews by qualified personnel at a call centre, or at the participating site if local regulations did not allow remote survey interviews via call centre. The decision on the collection approach was based on suggestions from patient experts and clinical experts and aimed to provide equal circumstances for the participants irrespective of the level of their visual impairment. Although we collected data from both patients and caregivers, participants could not enrol as both a patient and a caregiver to avoid potential bias in the results. Caregiver outcomes will be published separately.

The other source was retrospective data collected from patients’ medical records available at sites involved in the EXPLORE XLRP-2 study (i.e., ophthalmic centres routinely managing patients with IRDs). Retrospective data included patients’ socio-demographics, comorbidities, clinical parameters, date of first visit to participating site, diagnostic tools used, outcomes of the assessment, consultations, medical resource use, and up to 5 years of retrospective data, where available.

The cross-sectional patient surveys consisted of five patient-reported outcome (PRO) tools: 1. modified Low Luminance Questionnaire (mLLQ); 2. Work Productivity and Activity Impairment General Health v2.0 (WPAI-GH2); 3. Hospital Anxiety and Depression Scale (HADS); 4. Patient Global Impression (PGI)-Mobility; 5. PGI-Daily Activity; and a long-term impact patient questionnaire developed for the study (the outcomes of which will be published separately).

The mLLQ was a modified version of the original 32-item LLQ, an eye disease-specific questionnaire for assessing self-reported visual problems under low luminance and at night [14]. The mLLQ used in this study is the same as the one used in the “Phase 3 Randomized, Controlled Study of AAV5-hRKp.RPGR for the Treatment of X-linked Retinitis Pigmentosa Associated With Variants in the RPGR Gene” (https://clinicaltrials.gov/study/NCT04671433). Modifications were made to the LLQ based on patient feedback, with input from a clinical expert and instrument development experts. The reasons for the modifications included difficulty with interpretation, irrelevance, poor fit between response choice and the item, items assessing more than a single concept, difficulty selecting a response, and difficulty selecting between two response choices. The adult mLLQ, administered to individuals ≥18 years of age, consisted of 30 items across six domains: driving (4 items), extreme lighting (7 items), mobility (6 items), emotional distress (4 items), general dim lighting (6 items) and peripheral vision (3 items). The adolescent mLLQ, administered to individuals 12–17 years of age, comprised 22 items across five domains: extreme lighting (4 items), mobility (6 items), emotional distress (4 items), general dim lighting (5 items) and peripheral vision (3 items). Scores range from 0 (maximal difficulty) to 100 (no difficulty) for both adults and adolescents.

The WPAI-GH2 measured effects of general health and symptom severity on work productivity and regular activities [15]. It consisted of six questions: i. working for pay status (yes/no; if no, only question 6 was answered); ii. hours missed due to health problems; iii. hours missed for other reasons; iv. hours actually worked; v. degree to which health affected productivity while working (measured on a visual analogue scale from 0 to 10); and vi. degree to which health affected productivity in regular unpaid activities (visual analogue scale from 0 to 10). For the last two questions, the lowest score (0) represents no impact. The recall period for questions 2–6 was 7 days. Responses were used to derive four scores (absenteeism, presenteeism, work productivity loss and activity impairment). These four scores were expressed as impairment percentages (0–100%), with higher numbers indicating greater impairment/less productivity.

The HADS measured symptoms of anxiety and depression and comprised seven items, each with depression and anxiety subscales. Scoring for each item ranged from 0 to 3, with 3 denoting the highest anxiety or depression level. A total subscale score of >7 points out of a possible 21 denoted considerable symptoms of anxiety or depression [16, 17]. The recall period was 7 days.

The PGI-Mobility measured the impact of patients’ vision status on their mobility (e.g., walking outside, travelling, using stairs or curbs). The PGI-Daily Activity measured the impact of vision status on patients’ daily activities (e.g., seeing the television, recognising or meeting people, or reading) [18]. Each consisted of one item, and scoring ranged from ‘not at all’ to ‘very much’ on a 5-point scale. The recall period was 7 days.

Data were summarised using descriptive statistics. Continuous/ordinal variables were summarised using number of patients (n), mean, standard deviation (SD), median, minimum, maximum, and 95% confidence interval (CI). Categorical variables were summarised with n, percent and 95% CI. No truncation of negative lower CI value to 0 has taken place. Correlations between level of disease stage and level of burden were analysed exploratively using appropriate correlation methods, and all reported *p-*values were non-adjusted, nominal and exploratory in nature.

Analysis sets considered in this study included: (1) full analysis set (FAS): patients enrolled with data on at least one PRO and not enrolled as both patient and caregiver; (2) modified FAS (mFAS): a subset of FAS participants who did not discontinue the study for withdrawal reasons (withdrawal by legally authorised representative, withdrawal by parent or guardian, withdrawal by subject); and (3) modified per-protocol (mPP): a subset of mFAS participants who satisfied all inclusion and exclusion criteria.

Primary analysis was based on the mPP analysis set. Descriptive statistics are provided for the overall population as well as for each severity stage of XLRP. For ordinal variables, in addition to descriptive statistics, frequency distribution (number and percentage) is provided for each level of the ordinal variable by stage of XLRP. Correlation between stage of XLRP and continuous/ordinal variable was analysed and Kendall τ_b_ or τ_c_ rank correlation coefficient with corresponding *p-*values are provided, depending on the variable.

For the relationship of stage of XLRP with nominal variables (e.g., type of employment: full-time paid, part-time paid, etc.), frequency distributions (number and percentage with corresponding 95% CI) are provided for each category of categorical variable by stage of XLRP. Correlations between stage of XLRP and binomial variables (e.g., yes/no responses, such as ‘XLRP had impact on level of education’) were analysed and rank-biserial correlation coefficient (a measure of association estimated by Goodman–Kruskal’s gamma) with corresponding *p-*value is provided.

## Results

### Key patient demographics and baseline characteristics from medical records

Of 176 patients initially enrolled in EXPLORE XLRP-2, 169 were included in the mPP as four provided no PRO data, one withdrew consent, and one did not meet inclusion criteria. One participant was enrolled as both a patient and a caregiver, and the decision was made to include this participant as only a caregiver in the analyses (caregiver data will be published separately). One patient had no clinical-stage data, so was excluded from correlation analyses. Twenty were adolescents (12–17 years of age). The mean age was higher for patients with severe clinical stage compared with patients with mild stage (Table 1).

**Table 1:** Demographics of the mPP analysis set.

XLRP disease stage	Mild	Moderate	Severe	Total
N	62	44	62	169
**Mean age, years (SD)**	33.4 (16.76)	38.6 (15.25)	45.3 (17.87)	39.3 (17.61)
95% CI	29.1, 37.6	33.9, 43.2	40.8, 49.9	36.7, 42.0
**Male sex (%)**	41 (66.1%)	37 (84.1%)	58 (93.5%)	137 (81.1%)
95% CI	50.3, 79.0	66.3, 93.4	81.1, 98.0	72.5, 87.4
**Highest education level achieved**				
Did not complete secondary school or less than high school (%)	4 (6.5%)	6 (13.6%)	3 (4.8%)	13 (7.7%)
Some secondary or high school education (%)	7 (11.3%)	6 (13.6%)	0	13 (7.7%)
High school or secondary school degree complete (%)	3 (4.8%)	2 (4.5%)	10 (16.1%)	15 (8.9%)
Associate's or technical degree complete (%)	1 (1.6%)	2 (4.5%)	5 (8.1%)	8 (4.7%)
College or baccalaureate degree complete (%)	6 (9.7%)	6 (13.6%)	6 (9.7%)	18 (10.7%)
Doctoral or postgraduate education (%)	2 (3.2%)	2 (4.5%)	4 (6.5%)	8 (4.7%)
More than high school (%)	1 (1.6%)	1 (2.3%)	2 (3.2%)	4 (2.4%)
Special school attended (%)	0	0	0	0
Unknown (%)	38 (61.3%)	19 (43.2%)	32 (51.6%)	90 (53.3%)
**Ophthalmic history**				
N	27	23	33	83
Pseudophakia	5 (18.5%)	7 (30.4%)	14 (42.4%)	26 (31.3%)
High myopia	9 (33.3%)	5 (21.7%)	7 (21.2%)	21 (25.3%)
Cataract	9 (33.3%)	7 (30.4%)	6 (18.2)	22 (26.5)
Age at start: Mean (SD)	47.0 (17.46)	31.0 (10.18)	40.5 (10.15)	40.1 (14.78)
95% CI	33.6, 60.4	21.6, 40.4	29.8, 51.2	33.6, 46.7
**Age at confirmation of the diagnosis with genetic testing**				
N	62	44	60	167
Mean (SD)	28.0 (16.58)	32.5 (16.23)	39.2 (18.77)	33.4 (17.98)
95% CI	23.8, 32.2	27.5, 37.4	34.3, 44.0	30.7, 36.1
**Years from initial XLRP symptoms until genotyping results (based on dates)**				
N	55	39	56	150
Mean (SD)	9.4 (11.07)	13.3 (12.97)	25.5 (16.97)	16.4 (15.66)
95% CI	6.4, 12.3	9.1, 17.5	20.9, 30.0	13.9, 18.9

*CI* confidence interval, *mPP* modified per-protocol, *SD* standard deviation, *XLRP* X-linked retinitis pigmentosa.

The majority were male (81.1%). Females were reported to be mostly in the mild stage (Table 1). Approximately half (49.1%) of patients had any ophthalmic history in addition to XLRP, which included pseudophakia (31.3%), cataract (30.1%) and high myopia ( ≥ −6DS; 25.3%). Mean age (SD) at genetic confirmation was 33.4 years (17.98), with mean duration (SD) from initial symptoms to genetic diagnosis being 16.4 (15.66) years (Table 1) and mean (SD) time from sending the first genetic sample to getting results being 252.6 (361.50) days.

Overall, 66.3% of patients had known family members diagnosed with XLRP. The mean (SD) number of family members diagnosed with XLRP was 2.6 (1.50), with 1.7 (1.41) family members confirmed by genetic testing.

### Outcomes from patient self-reported surveys

#### mLLQ

**Fig. 1:**
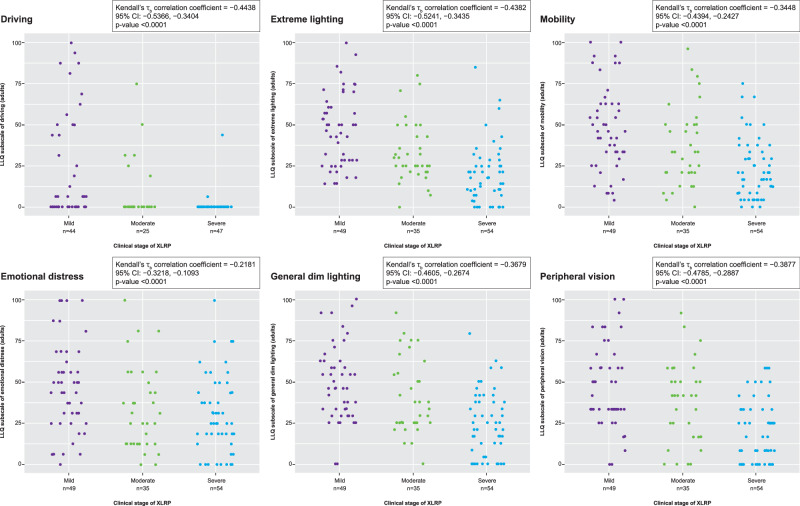
Modified LLQ outcomes. A score of 0 indicates maximal difficulty, and a score of 100 indicates no difficulty. LLQ Low Luminance Questionnaire, XLRP X-linked retinitis pigmentosa.

The mLLQ results revealed that adult patients with severe disease experience greater difficulties functioning in low luminance and at night compared with patients with mild disease. For adults, all six domains were significantly negatively correlated with clinical stage (Fig. 1; Supplementary Table 2). For adolescents, similar trends were observed, but only three domains were significantly negatively correlated with disease stage: mobility, general dim lighting and peripheral vision (Supplementary Table 3).

#### HADS

On the HADS questionnaire, 28.0% of patients reported any level of depression and 44.6% reported any level of anxiety (Table 2).

**Table 2:** HADS scores by clinical stage in the mPP analysis set.

XLRP disease stage	Mild	Moderate	Severe	Total
*N*	58	40	59	157
Depression				
Normal (%)	46 (79.3%)	26 (65.0%)	41 (69.5%)	113 (72.0%)
Mild (%)	10 (17.2%)	5 (12.5%)	9 (15.3%)	24 (15.3%)
Moderate (%)	2 (3.4%)	7 (17.5%)	7 (11.9%)	16 (10.2%)
Severe (%)	0	2 (5.0%)	2 (3.4%)	4 (2.5%)
Mean score (SD)	4.00 (3.27)	5.50 (5.10)	5.80 (3.93)	5.06 (4.10)
95% CI	3.14, 4.86	3.87, 7.13	4.77, 6.82	4.42, 5.71
Kendall’s τ_b_	0.1551			
95% CI	0.0515, 0.2553			
*p*-value	0.0034			
Anxiety				
Normal (%)	36 (62.1%)	23 (57.5%)	28 (47.5%)	87 (55.4%)
Mild (%)	11 (19.0%)	8 (20.0%)	17 (28.8%)	36 (22.9%)
Moderate (%)	8 (13.8%)	6 (15.0%)	12 (20.3%)	26 (16.6%)
Severe (%)	3 (5.2%)	3 (7.5%)	2 (3.4%)	8 (5.1%)
Mean score (SD)	6.66 (4.14)	8.00 (4.37)	7.34 (4.17)	7.25 (4.21)
95% CI	5.57, 7.74	6.60, 9.40	6.25, 8.42	6.58, 7.90
Kendall’s τ_b_	0.0837			
95% CI	−0.0209, 0.1864			
*p*-value	0.1166			

*CI* confidence interval, *HADS* Hospital Anxiety and Depression Scale, *mPP* modified per-protocol, *SD* standard deviation, *XLRP* X-linked retinitis pigmentosa.

Correlation of HADS depression measures with XLRP stage was significant, as patients with severe disease were more likely to report any level of depression compared with patients with moderate or mild disease. However, mean scores (overall and in any stage) did not reach the depression threshold defined by the scale.

Correlation of HADS anxiety measures with XLRP stage was not significant. However, mean overall scores were slightly above the anxiety threshold defined by the scale. Moreover, both moderate and severe patients tended to have higher mean anxiety scores and lower proportions of patients without any anxiety than patients with mild disease.

#### WPAI-GH2

The WPAI-GH2 was administered to adult patients (*n* = 148). 138 completed the questionnaire, with 76 (55.1%) employed. Patients with more severe disease were less likely to be employed, but the correlation was not significant (Supplementary Table 4).

**Fig. 2:**
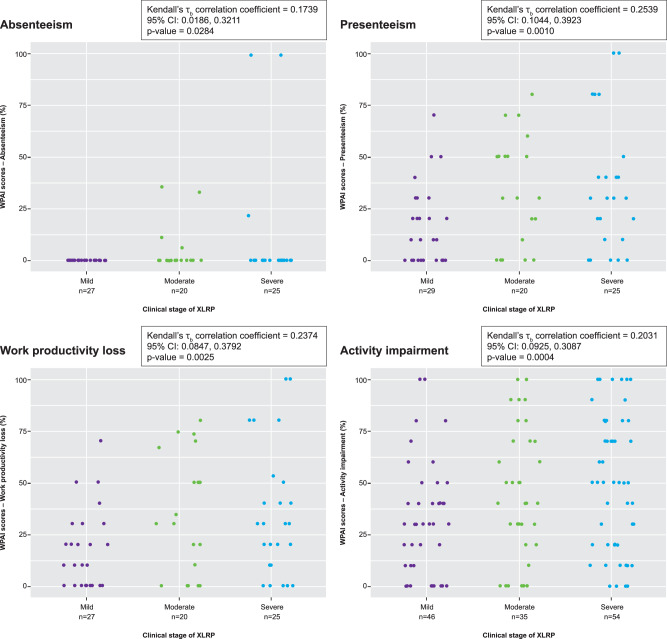
WPAI-GH2 outcomes. Scores were expressed as impairment percentages (0–100%), with higher numbers indicating greater impairment/less productivity. WPAI-GH2 Work Productivity and Activity Impairment General Health v2.0, XLRP X-linked retinitis pigmentosa.

For employed patients, WPAI-GH2 data showed absenteeism, impairment while working (presenteeism) and work productivity loss all correlated significantly with XLRP stage, with patients with severe XLRP tending to miss more days at work and to experience greater work impairment and productivity loss compared with patients with mild disease. Absenteeism was low for all patients, and patients with mild-stage XLRP reported no absenteeism at all. Activity impairment scores also correlated significantly with clinical stage (Fig. 2; Supplementary Table 4).

#### PGI

PGI-Mobility and PGI-Daily Activity scores significantly correlated with disease stages (Table 3; Supplementary Fig. 1), with patients with severe disease more likely to report more impacts than patients with mild or moderate disease. Notably, two-thirds of patients with mild disease reported any level of impact on mobility and daily activities. These impacts were reported in >90% of patients in the severe stage of disease.

**Table 3:** Summary of PGI questionnaire responses in the mPP analysis set.

XLRP disease stage	Mild	Moderate	Severe	Total
*N*	58	40	59	157
PGI-Mobility				
Not at all	17 (29.3%)	7 (17.5%)	4 (6.8%)	28 (17.8%)
A little bit	18 (31.0%)	7 (17.5%)	8 (13.6%)	33 (21.0%)
Somewhat	17 (29.3%)	10 (25.0%)	17 (28.8%)	44 (28.0%)
Quite a bit	3 (5.2%)	9 (22.5%)	11 (18.6%)	23 (14.6%)
Very much	3 (5.2%)	7 (17.5%)	19 (32.2%)	29 (18.5%)
Mean score (SD)	2.26 (1.10)	3.05 (1.36)	3.56 (1.26)	2.95 (1.35)
Median	2.00	3.00	4.00	3.00
95% CI	1.97, 2.55	2.62, 3.48	3.23, 3.89	2.74, 3.17
Kendall’s τ_b_	0.3831			
95% CI	0.2624, 0.5038			
*p*-value	<0.0001			
PGI-daily activity				
Not at all	21 (36.2%)	6 (15.0%)	4 (6.8%)	31 (19.7%)
A little bit	16 (27.6%)	9 (22.5%)	9 (15.3%)	34 (21.7%)
Somewhat	12 (20.7%)	7 (17.5%)	8 (13.6%)	27 (17.2%)
Quite a bit	6 (10.3%)	6 (15.0%)	10 (16.9%)	22 (14.0%)
Very much	3 (5.2%)	12 (30.0%)	28 (47.5%)	43 (27.4%)
Mean score (SD)	2.21 (1.20)	3.23 (1.48)	3.83 (1.35)	3.08 (1.50)
Median	2.00	3.00	4.00	3.00
95% CI	1.89, 2.52	2.75, 3.70	3.48, 4.18	2.85, 3.32
Kendall’s τ_b_	0.4307			
95% CI	0.3137, 0.5477			
*p*-value	<0.0001			

*CI* confidence interval, *mPP* modified per-protocol, *PGI* Patient Global Impression, *SD* standard deviation, *XLRP* X-linked retinitis pigmentosa.

## Discussion

To our knowledge, this is the first real-world study to collect data directly from patients on the burden of XLRP and to correlate that burden with disease stage. Despite XLRP being a rare eye disease, we surveyed 169 patients across Europe and Israel to understand their experiences of living with the disease.

Medical records showed that, on average, over 15 years elapsed between symptom onset and genetic diagnosis. However, shorter durations in patients with mild disease suggest these delays have improved over time. This is not surprising, as technologies improve and access to genetic testing becomes more widespread [19, 20]. Early genetic confirmation of IRDs reduces unnecessary examinations, thus cutting medical costs and improving patient well-being, while also improving eligibility for prospective gene-based treatments [20]. Therefore, genetic confirmation of XLRP at a mean age of 33.4 years is too late. Increasing awareness of IRDs and importance of genetic testing through IRD-specific next-generation sequencing panels will help deliver earlier relevant diagnoses.

We also demonstrated that XLRP exerts a significant impact on patients. As expected, patients with severe disease tended to face the greatest burdens, with visual problems in low luminance, work time missed, impairment while working, work productivity loss, mobility and daily activities all significantly correlating with disease stage. Moreover, PGI-Mobility and PGI-Daily Activity results indicated that even patients with mild disease are already considerably impacted.

In our study, 28% of patients reported any level of depression and 44.6% reported any level of anxiety. Our proportions are higher than those reported by Chaumet-Riffaud et al., who applied HADS to patients with RP in France (depression: 15.5%; anxiety: 36.5%), suggesting the more severe XLRP has a higher impact relative to other RPs [21]. For HADS depression, our measures significantly correlated with XLRP stage. Chaumet-Riffaud et al. found a non-significant trend for RP [21]. However, Sainohira et al., who applied HADS to patients with RP in Japan, did find a significant correlation [22]. For HADS anxiety, we found the proportion of patients who reported any level of anxiety tended to be greater in the moderate and severe disease stages, but this was not a significant correlation. Similarly, neither Chaumet-Riffaud et al. nor Sainohira et al. found correlations between degree of visual impairment and anxiety in patients with RP [21, 22]. Interestingly, Sainohira et al. also found that depression was lower in employed than unemployed visually impaired patients [22].

The WPAI questionnaire revealed that just over half of patients were employed, a statistic mirrored by a 2014 population study in the United States, which found that 58.7% of men with visual impairment were employed, compared with 76.2% of men with normal vision [23]. That study also found that decreased vision was associated with a higher likelihood of unemployment, whereas we did not find a significant correlation between disease stage and working status [23]. Our findings likely reflect the effectiveness of regulations on facilitated employment for visually impaired people from the surveyed countries in supporting XLRP patients who are motivated to continue their working life. A targeted literature review by Galvin et al. of data pertaining to the burden of IRDs in the United Kingdom (UK) and in the Republic of Ireland also found that, overall, nearly half (45.5%) of participants with IRD were employed, compared with 76.1% of the general population [24]. Of note, our study found absenteeism was low for all disease stages and zero for patients with mild disease, similar to the finding by Galvin et al. of zero absenteeism for patients with IRD in the UK and Ireland [24]. Beyond the obvious financial benefits, employment can have emotional and psychological benefits [23]. The degree of support from employers may be reflected in the low observed levels of absenteeism among employed patients. Patients who do not feel supported by their employer may avoid taking time off, potentially increasing negative impacts on productivity.

As discussed in studies with similar methodologies [11], we acknowledge this study may have bias against patients with severe anxiety and depression. Such individuals may not participate, or they may not be asked to participate by physicians. This might shift questionnaire outcomes to patients with more positive mindsets, higher education and more secure employment than the true average of the affected patients.

Another limitation is the relatively short recall period of the PRO tools employed, which typically had a recall period of 7 days and reflected the current disease status rather than long-term impact of this chronic disease. In addition, the study lacks an analysis of refraction data, as only BCVA data were analysed to assess visual acuity with refraction errors. Finally, the small sample size of female participants per stage is a limitation, preventing robust subgroup analysis. Further research is recommended to enhance the understanding of the impact of gender on the relationship between the clinical stage of XLRP and the associated clinical, individual and societal levels of burden.

The outcomes of this study offer valuable insights into the impacts of XLRP on many aspects of daily life. Our findings highlight unmet needs of patients with XLRP, who are faced with significant emotional and societal burdens that increase as the disease progresses. Understanding these needs is critical for development of novel therapies and management strategies for XLRP. Further studies that continue to explore the patient experience of living with XLRP are needed to deepen our understanding of humanistic impacts of this disease, particularly across other regions.

Supplementary information is available at *Eye*’s website.

## Summary

### What was known before

- XLRP is a severe, progressive retinal disease that impacts vision at an early age and ultimately leads to blindness.
- Patients with visual impairments often face a diverse array of burdens on their everyday lives, yet the specific impacts of XLRP are unknown.

### What this study adds

- This is the first study to evidence the complex burden of XLRP linked to disease stages.
- Despite improvements in genetic technologies, patients with XLRP still experience an average delay of more than 15 years from symptom onset to genetic diagnosis.
- Patients with XLRP face emotional and societal burdens, many of which significantly correlate with disease stage. These results provide areas of focus for research into disease management, for improving clinical practice, and for updating public policies that provide support for patients with XLRP.

## Supplementary information

Supplementary Figure 1

Supplemental File

## Data Availability

The datasets generated and/or analysed during the current study are available from the corresponding author upon reasonable request.
