# Supplementary figures and images for: The burden of X-linked retinitis pigmentosa (XLRP) on patient experience and patient-reported outcomes (PROs): findings from the EXPLORE XLRP-2 study
Source: Eye (Lond). 2025 Jan 7;39(3):578–85. doi: 10.1038/s41433-024-03546-8 (PMC11794432; doi:10.1038/s41433-024-03546-8)

Mobility

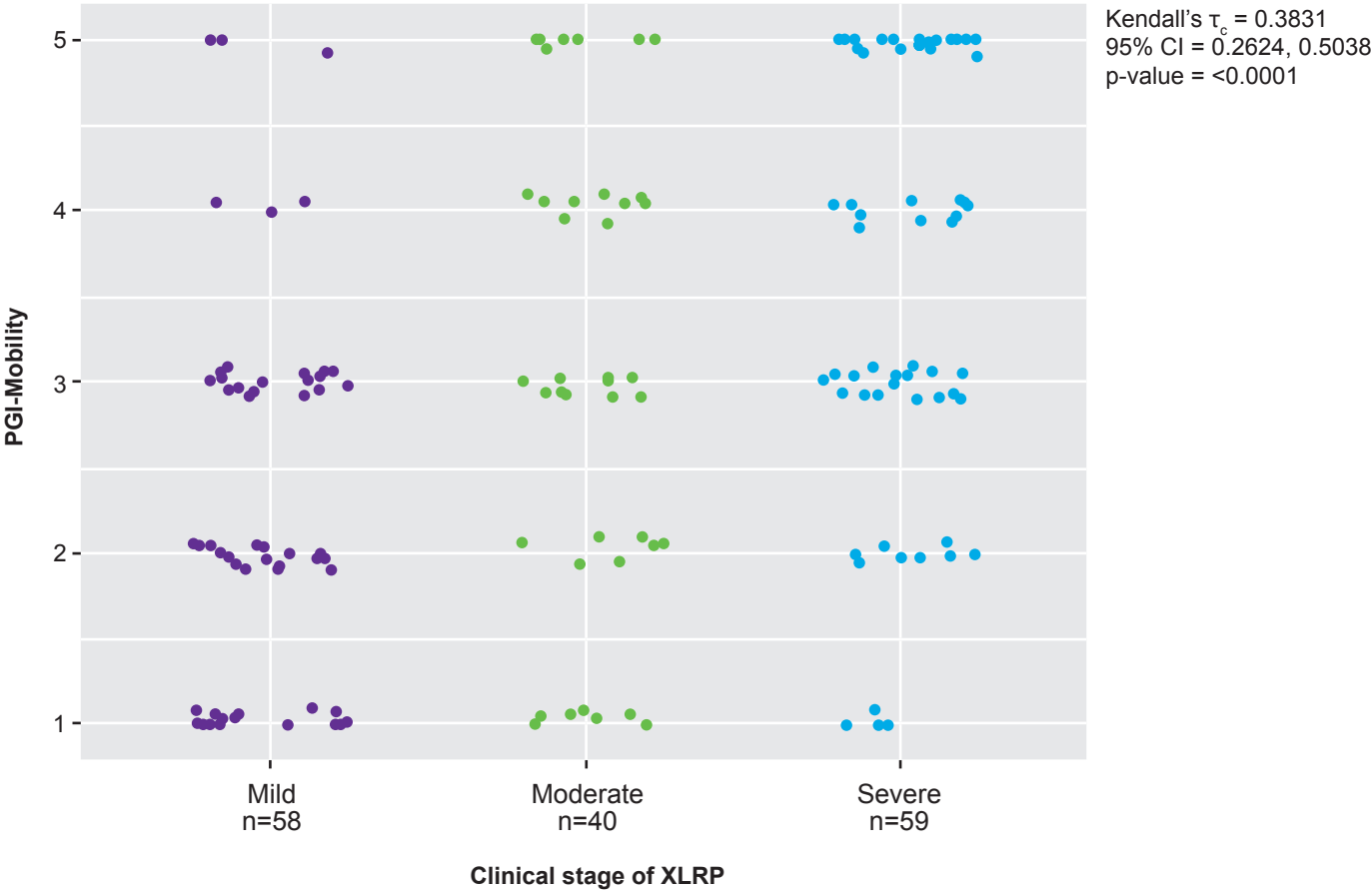

Daily activities

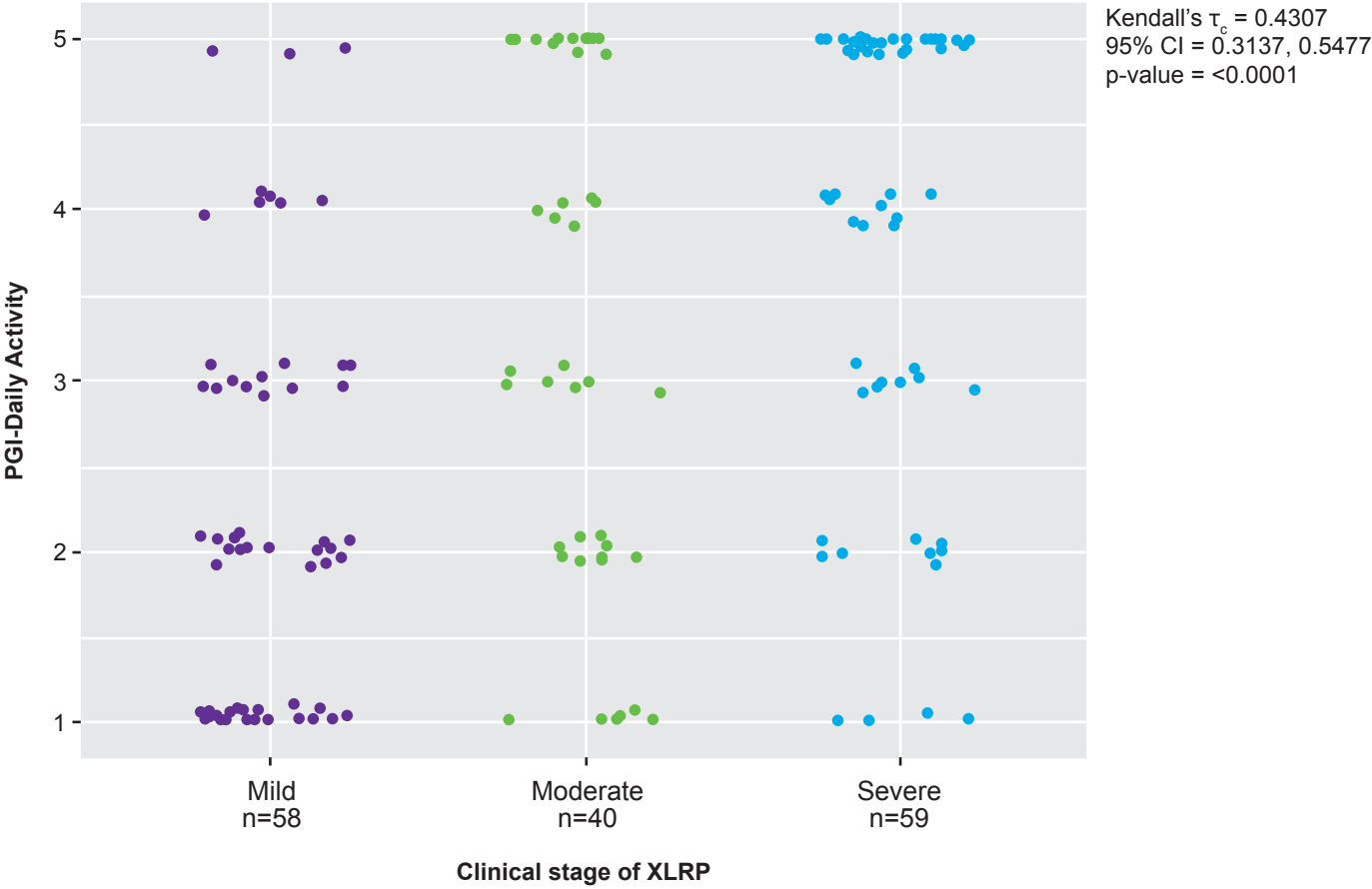

Supplement: Supplementary file 1 — Supplementary Figure 1 [file 41433_2024_3546_MOESM1_ESM.pdf]
