# Supplementary material for: The burden of X-linked retinitis pigmentosa (XLRP) on patient experience and patient-reported outcomes (PROs): findings from the EXPLORE XLRP-2 study
Source: Eye (Lond). 2025 Jan 7;39(3):578–85. doi: 10.1038/s41433-024-03546-8 (PMC11794432; doi:10.1038/s41433-024-03546-8)
Supplement: Supplementary file 2 — Supplemental File [file 41433_2024_3546_MOESM2_ESM.docx]

# SUPPLEMENTARY INFORMATION

**The burden of X-linked retinitis pigmentosa (XLRP) on patient experience and patient-reported outcomes (PROs): findings from the EXPLORE XLRP-2 study**

**Running title:** X-linked retinitis pigmentosa burden on patients

**Authors:** Francesco Parmeggiani*^1,2^, Michel Weber*^3^, Dominique Bremond-Gignac^4^, Avril Daly^5^, Tom Denee^6^, Marjolein Lahaye^6^, Andrew Lotery^7^, Nabin Paudel^5^, Markus Ritter^8^, Enrique Rodríguez de la Rúa^9^, Ygal Rotenstreich^10^, Eeva-Marja Sankila^11^, Katarina Stingl^12^, Jacqueline Van Denderen^6^, Katalin Pungor^13^

*Co-first authors

**Corresponding author:** Katalin Pungor, MD, PhD, MBA, Regional Medical Director, J&J Innovative Medicine, Europe Middle East and Africa (EMEA Neuss, Germany)

Tel: +49 1724 463940; Email: kpungor@its.jnj.com

**Supplementary Table 1.** Classification of XLRP disease stages

**Supplementary Table 2.** Modified LLQ domain statistics for adult patients

**Supplementary Table 3.** Modified LLQ domain statistics for adolescent patients

**Supplementary Table 4.** WPAI outcomes

**Supplementary Figure 1.** PGI outcomes

## Supplementary Table 1. Classification of XLRP disease stages

| **XLRP disease stage** | **Visual acuity** | | **Visual field** |
| --- | --- | --- | --- |
|  | Decimal notation | Snellen | Diameter |
| Mild | ≥0.32 | ≥20/63 | ≥80° |
| Moderate | <0.32, ≥0.125 | <20/63, ≥20/160 | <80°, ≥40° |
| Severe | <0.125 | <20/160 | <40° |

XLRP, X-linked retinitis pigmentosa.

## Supplementary Table 2. Modified LLQ domain statistics for adult patients

| **XLRP disease stage** | **Mild** | **Moderate** | **Severe** | **Total** |
| --- | --- | --- | --- | --- |
| **Driving** | | | | |
| N | 44 | 25 | 47 | 116 |
| Mean (SD) | 23.86 (31.85) | 9.25 (19.27) | 1.06 (6.43) | 11.48 (24.03) |
| 95% CI | 14.18, 33.55 | 1.29, 17.21 | −0.82, 2.95 | 6.99, 15.76 |
| Kendall’s τ_b_ | −0.4438 | | | |
| 95% CI | −0.5366, −0.3404 | | | |
| *p-*value | <0.0001 | | | |
| **Extreme lighting** | | | | |
| N | 49 | 35 | 54 | 138 |
| Mean (SD) | 47.36 (22.26) | 32.74 (18.35) | 20.14 (17.50) | 33.00 (22.68) |
| 95% CI | 40.97, 53.75 | 26.43, 39.04 | 15.36, 24.91 | 28.94, 36.58 |
| Kendall’s τ_b_ | −0.4382 | | | |
| 95% CI | −0.5241, −0.3435 | | | |
| *p-*value | <0.0001 | | | |
| **Mobility** | | | | |
| N | 49 | 35 | 54 | 138 |
| Mean (SD) | 47.36 (25.70) | 36.76 (23.89) | 23.07 (18.47) | 35.17 (24.83) |
| 95% CI | 39.98, 54.74 | 28.55, 44.97 | 18.03, 28.11 | 30.81, 39.14 |
| Kendall’s τ_b_ | −0.3448 | | | |
| 95% CI | −0.4394, −0.2427 | | | |
| *p-*value | <0.0001 | | | |
| **Emotional distress** | | | | |
| N | 49 | 35 | 54 | 138 |
| Mean (SD) | 47.83 (27.41) | 32.68 (25.55) | 30.56 (24.21) | 37.23 (26.74) |
| 95% CI | 39.96, 55.70 | 23.90, 41.46 | 23.95, 37.16 | 32.52, 41.50 |
| Kendall’s τ_b_ | −0.2181 | | | |
| 95% CI | −0.3218, −0.1093 | | | |
| *p-*value | 0.0001 | | | |
| **General dim lighting** | | | | |
| N | 49 | 35 | 54 | 138 |
| Mean (SD) | 48.38 (22.96) | 40.74 (22.36) | 23.77 (19.54) | 36.81 (23.99) |
| 95% CI | 41.79, 54.98 | 33.06, 48.42 | 18.43, 29.10 | 32.50, 40.59 |
| Kendall’s τ_b_ | −0.3679 | | | |
| 95% CI | −0.4605, −0.2674 | | | |
| *p-*value | <0.0001 | | | |
| **Peripheral vision** | | | | |
| N | 49 | 35 | 54 | 138 |
| Mean (SD) | 48.30 (25.51) | 38.10 (23.85) | 20.06 (18.35) | 34.66 (25.56) |
| 95% CI | 40.97, 55.63 | 29.90, 46.29 | 15.05, 25.07 | 30.11, 38.71 |
| Kendall’s τ_b_ | −0.3877 | | | |
| 95% CI | −0.4785, −0.2887 | | | |
| *p-*value | <0.0001 | | | |

CI, confidence interval; LLQ, Low Luminance Questionnaire; SD, standard deviation; XLRP, X-linked retinitis pigmentosa.

## Supplementary Table 3. Modified LLQ domain statistics for adolescent patients

| **XLRP disease stage** | **Mild** | **Moderate** | **Severe** | **Total** |
| --- | --- | --- | --- | --- |
| N | 9 | 5 | 5 | 19 |
| **Extreme lighting** | | | | |
| Mean (SD) | 70.83 (20.73) | 73.75 (10.27) | 57.50 (12.02) | 68.09 (17.04) |
| 95% CI | 54.90, 86.77 | 61.00, 86.50 | 42.57, 72.43 | 59.88, 76.30 |
| Kendall’s τ_b_ | −0.2819 | | | |
| 95% CI | −0.5541, 0.0448 | | | |
| *p-*value | 0.0896 | | | |
| **Mobility** | | | | |
| Mean (SD) | 71.30 (20.03) | 46.67 (11.18) | 31.67 (11.26) | 54.39 (23.18) |
| 95% CI | 55.90, 86.69 | 32.78, 60.55 | 17.69, 45.64 | 43.21, 65.56 |
| Kendall’s τ_b_ | −0.6369 | | | |
| 95% CI | −0.7960, −0.3956 | | | |
| *p-*value | <0.0001 | | | |
| **Emotional distress** | | | | |
| Mean (SD) | 56.25 (24.80) | 71.25 (7.13) | 38.75 (26.30) | 55.59 (24.20) |
| 95% CI | 37.18, 75.32 | 62.40, 80.10 | 6.10, 71.40 | 43.93, 67.25 |
| Kendall’s τ_b_ | −0.1029 | | | |
| 95% CI | −0.4118, 0.2272 | | | |
| *p-*value | 0.5452 | | | |
| **General dim lighting** | | | | |
| Mean (SD) | 68.89 (18.84) | 52.00 (12.55) | 38.00 (5.70) | 56.32 (19.43) |
| 95% CI | 54.41, 83.37 | 36.42, 67.58 | 30.92, 45.08 | 46.95, 65.68 |
| Kendall’s τ_b_ | −0.5786 | | | |
| 95% CI | −0.7594, −0.3148 | | | |
| *p-*value | 0.0001 | | | |
| **Peripheral vision** | | | | |
| Mean (SD) | 68.52 (18.06) | 53.33 (16.25) | 35.00 (27.26) | 55.70 (23.90) |
| 95% CI | 54.64, 82.40 | 33.16, 73.50 | 1.15, 68.85 | 44.18, 67.22 |
| Kendall’s τ_b_ | −0.4719 | | | |
| 95% CI | −0.6895, −0.1761 | | | |
| *p-*value | 0.0027 | | | |

CI, confidence interval; LLQ, Low Luminance Questionnaire; SD, standard deviation; XLRP, X-linked retinitis pigmentosa.

## Supplementary Table 4. WPAI outcomes

| **XLRP disease stage** | **Mild** | **Moderate** | **Severe** | **Total** |
| --- | --- | --- | --- | --- |
| **Current employment status** |  |  |  |  |
| N | 49 | 35 | 54 | 138 |
| No | 18 (36.7%) | 15 (42.9%) | 29 (53.7%) | 62 (44.9%) |
| 95% CI | 23.4, 51.7 | 26.3, 60.6 | 39.6, 67.4 | 36.5, 53.6 |
| Yes | 31 (63.3%) | 20 (57.1%) | 25 (46.3%) | 76 (55.1%) |
| 95% CI | 48.3, 76.6 | 39.4, 73.7 | 32.6, 60.4 | 46.4, 63.5 |
| Correlation with clinical stage | −0.2426 | | | |
| 95% CI | −0.5060, 0.0209 | | | |
| *p-*value | 0.0779 | | | |
| **Absenteeism (%)** | | | | |
| N | 27 | 20 | 25 | 72 |
| Mean (SD) | 0.0 (0.00) | 4.3 (10.73) | 8.9 (27.77) | 4.3 (17.49) |
| 95% CI | 0.0, 0.0 | −0.7, 9.4 | −2.6, 20.3 | 0.2, 8.4 |
| Kendall’s τ_b_ | 0.1739 | | | |
| 95% CI | 0.0186, 0.3211 | | | |
| *p-*value | 0.0284 | | | |
| **Presenteeism (%)** | | | | |
| N | 29 | 20 | 25 | 74 |
| Mean (SD) | 15.2 (18.83) | 33.5 (26.61) | 34.8 (31.11) | 26.8 (26.95) |
| 95% CI | 8.0, 22.3 | 21.0, 46.0 | 22.0, 47.6 | 20.5, 33.0 |
| Kendall’s τ_b_ | 0.2539 | | | |
| 95% CI | 0.1044, 0.3923 | | | |
| *p-*value | 0.0010 | | | |
| **Work productivity loss (%)** | | | | |
| N | 27 | 20 | 25 | 72 |
| Mean (SD) | 16.3 (19.04) | 35.4 (28.49) | 35.3 (31.31) | 28.2 (27.71) |
| 95% CI | 8.8, 23.8 | 22.1, 48.8 | 22.4, 48.2 | 21.7, 34.7 |
| Kendall’s τ_b_ | 0.2374 | | | |
| 95% CI | 0.0847, 0.3792 | | | |
| *p-*value | 0.0025 | | | |
| **Activity impairment (%)** | | | | |
| N | 46 | 35 | 54 | 135 |
| Mean (SD) | 30.9 (27.23) | 44.9 (31.09) | 49.3 (32.55) | 41.9 (31.30) |
| 95% CI | 22.8, 39.0 | 34.2, 55.5 | 40.4, 58.1 | 36.5, 47.2 |
| Kendall’s τ_b_ | 0.2031 |  |  |  |
| 95% CI | 0.0925, 0.3087 |  |  |  |
| *p-*value | 0.0004 |  |  |  |

CI, confidence interval; SD, standard deviation; XLRP, X-linked retinitis pigmentosa; WPAI, Work Productivity and Activity Impairment.

## Supplementary Figure 1. PGI outcomes

Scoring ranged from ‘not at all’ to ‘very much’ on a 5-point scale.

CI, confidence interval; PGI, Patient Global Impression; XLRP, X-linked retinitis pigmentosa.
